# Knowledge, Confidence and Preparedness for Brain Death Determination Among Anaesthesiology and Reanimation Residents: A National Web-Based Survey with an Embedded Randomised Vignette

**DOI:** 10.3390/healthcare14203495

**Published:** 2026-10-17

**Authors:** Seher Yanatma, Sedat Kaya

**Affiliations:** 1 Department of Intensive Care, Gazi Yaşargil Training and Research Hospital, University of Health Sciences, 21070 Diyarbakır, Turkey; 2 Department of Anaesthesiology and Reanimation, Gazi Yaşargil Training and Research Hospital, University of Health Sciences, 21070 Diyarbakır, Turkey

**Keywords:** brain death, death by neurologic criteria, graduate medical education, self-assessment, self-efficacy, clinical decision-making, organ donation, survey, Türkiye

## Abstract

Background/Objectives: Under Turkish law, brain death is determined by two physicians, one of whom is a specialist in anaesthesiology and reanimation or intensive care, a responsibility residents assume soon after qualifying. We assessed whether residents’ confidence matches their accuracy, and whether the decision to initiate determination is influenced by the family’s attitude toward organ donation. Methods: Cross-sectional web-based survey of a national convenience sample of anaesthesiology and reanimation residents in Türkiye. Ten knowledge statements and three clinical vignettes combined the answer and the respondent’s confidence in one choice; the primary outcome was the high-confidence error rate. In an embedded participant-blinded randomised vignette, participants were allocated 1:1 to a medically identical scenario describing the family as favourable or opposed to donation. Results: A total of 206 residents were analysed. They answered 80.9% of items correctly but endorsed only 64.2% with confidence; confident answers were correct in 90.8% of instances versus 63.3% of non-confident answers (*p* < 0.001). The mean high-confidence error rate was 5.9 ± 7.3%, yet 50.5% committed at least one such error, and these errors clustered in four domains. In this exploratory comparison, initiation of determination did not differ between arms (85.6% versus 87.4%; adjusted odds ratio 1.29, 95% CI 0.54–3.05). Only 18.0% felt prepared to serve on a determination board; in univariable and sensitivity analyses, preparedness was associated with case exposure and formal instruction but not with knowledge score. Conclusions: Residents answered most items correctly and were appropriately cautious, yet fewer than one in five felt prepared; self-reported preparedness is not a measure of clinical competence. Supervised clinical exposure, correction of specific misconceptions and communication training are priorities for future intervention studies.

## 1. Introduction

Brain death is the irreversible cessation of all functions of the entire brain, including the brainstem, and is legally and medically equivalent to death [1,2]. Its determination is among the most consequential decisions made in intensive care: it establishes the time of death, ends life-sustaining therapy, and—when the family so chooses—opens the possibility of organ donation. In Türkiye, the determination of brain death is governed by Law No. 2238 on the Procurement, Storage, Grafting and Transplantation of Organs and Tissues [3]. Under the current regulation, the diagnosis is established by the unanimous decision of two physicians: one neurologist or neurosurgeon, and one specialist in anaesthesiology and reanimation or in intensive care medicine. Consequently, every physician completing residency training in anaesthesiology and reanimation may, within a few years, become one of the two signatories to this determination. The competence of today’s residents is therefore not an abstract educational concern but a direct determinant of the quality of a legally binding clinical decision. Several surveys have documented deficiencies in physicians’ knowledge of brain death criteria. In a web-based survey of physicians working predominantly in critical care settings, Vasconcelos and colleagues found knowledge of determination protocols to be frequently inadequate and, counterintuitively, reported that greater intensive care experience was not associated with better performance [4]. In the national context, Mutlu and Utku surveyed anaesthesiology and reanimation professionals in Türkiye and documented comparable gaps [5]. A web-based survey of clinicians working in Turkish intensive care units likewise identified physician hesitation and insufficient education as the principal impediments to diagnosis, even though most respondents rated their own competence as sufficient [6]. Substantial variation in determination protocols between and within countries has also been described [7], as has variation in institutional policies and in the practices that physicians report at the bedside [8,9,10]. These studies share a common design: they report the proportion of correct answers. Yet, from the standpoint of patient safety, the more consequential quantity is not how much a clinician knows, but how accurately that clinician knows the limits of their own knowledge. A physician who recognises uncertainty will seek supervision, consult a colleague, or defer the examination. A physician who holds an incorrect belief with high subjective certainty will act on it. The mismatch between confidence and accuracy—miscalibration—has been characterised in other clinical domains, where overconfidence has been proposed as a contributor to diagnostic error [11] and where physicians’ certainty has been shown to correspond only loosely to the accuracy of their conclusions [12]. More broadly, self-assessment is an unreliable guide to competence: those who perform least well tend to overestimate themselves most [13], and physicians’ self-ratings correspond poorly to externally observed measures of competence [14,15]. To our knowledge, miscalibration has not been examined in the context of brain death determination. A second question concerns the integrity of the determination process itself. International guidance and national regulation alike require that the determination of brain death be made on medical grounds alone, entirely independently of any prospect of organ donation, and that the determining team be separate from the donation coordination team [1,3]. Whether this separation is preserved in the clinical reasoning of physicians in training has not been tested experimentally. This study was designed to address both questions. The primary objective was to quantify the rate of high-confidence errors—responses that were both incorrect and endorsed with subjective certainty—in brain death determination among anaesthesiology and reanimation residents. Secondary objectives were: (i) to identify the knowledge domains in which such errors concentrate; (ii) to determine whether high-confidence error is associated with formal instruction or with accumulated case exposure; (iii) to test, by means of an embedded randomised vignette, whether the decision to initiate the determination process is influenced by the family’s stated attitude toward organ donation in an otherwise medically identical patient; and (iv) to describe residents’ perceived preparedness to undertake this responsibility, together with the barriers they anticipate.

## 2. Materials and Methods

### 2.1. Study Design and Reporting

This was a cross-sectional web-based survey of a national convenience sample of residents, with an embedded, participant-blinded, two-arm randomised clinical vignette. The study was conducted in accordance with the principles of the Declaration of Helsinki. Reporting follows the Checklist for Reporting Results of Internet E-Surveys (CHERRIES) [16] and, where applicable, the STROBE statement for cross-sectional studies [17]. The embedded vignette experiment allocated physicians rather than patients and assessed no health-related outcome; it therefore does not meet the definition of a clinical trial and was not registered.

### 2.2. Ethical Approval and Consent

The protocol was approved by the Clinical Research Ethics Committee of the University of Health Sciences, Gazi Yaşargil Training and Research Hospital, Diyarbakır, Türkiye (decision no. 420, dated 28 August 2026). The approved protocol specified that the existence of two vignette versions would not be disclosed until the debriefing screen described below. Participation was voluntary and anonymous. No names, email addresses, IP addresses or other identifying information were collected. Before the first question, participants were shown an information text and provided digital informed consent; those who declined were excluded from the survey. The information text stated explicitly that individual scores would not be reported to participants or to any third party, that results would not be reported at the level of the individual or the institution, and that the data would under no circumstances be used for performance appraisal. Because responses were fully anonymous, participants were informed that withdrawal of data after submission would not be technically possible.

### 2.3. Participants and Recruitment

Eligible participants were physicians currently enrolled in residency training in anaesthesiology and reanimation in Türkiye, aged 18 years or older. Specialists, faculty members, residents in other disciplines, medical students, and general practitioners were not eligible. Respondents who did not provide consent left the survey at the consent screen and were not included in the analysis. The survey link was distributed through professional networks of anaesthesiology and reanimation residents and through departmental and clinic chairs at university, training, and research hospitals. Because the survey was anonymous and the name of the institution was not collected, the number of participating centres cannot be stated. The sample is therefore a national convenience sample: residents were recruited across the country, but not by any probability-based method, and the sample is not intended to be representative of all anaesthesiology and reanimation residents in Türkiye. The form did not require sign-in, so multiple submissions by one individual could not be technically prevented; the dataset was screened for identical response records and none was found. Data were collected between 31 August and 20 September 2026. Participation was voluntary and no incentives were offered.

### 2.4. Instrument Development

The questionnaire was developed by the two authors—an intensive care specialist and a specialist in anaesthesiology and reanimation—on the basis of the current national regulation [3] and contemporary international guidance on brain death determination [1,2]. The answer key followed the national regulation in force (Annex 1 of the Regulation on Organ and Tissue Transplantation Services) [3]. Where national and international requirements differ—notably the interval between the two neurological examinations and the carbon dioxide criterion of the apnoea test—the national requirement was keyed as correct, since it is the standard to which respondents are legally held. Content validity was assessed only by the authors’ review and by pretesting with specialists. Both authors reviewed each item against the national regulation and the international guidance for clinical accuracy, relevance and clarity of wording, and the electronic form was tested for functionality and readability on desktop and mobile devices before distribution. The draft questionnaire was then pretested with 20 specialists in anaesthesiology and reanimation, who were not part of the target population. The specialists completed the electronic form in full, so that its operation could be checked, and commented on the clarity, clinical accuracy and relevance of the items; no changes to the items were required. Their responses served only to verify the functioning of the form, were not analysed, and were deleted before data collection began. No quantitative assessment of content validity (such as a content validity index), no pilot administration in the target population and no formal psychometric validation were undertaken. The full instrument, including both versions of the randomised vignette, is provided as Supplementary File S1. The instrument was purpose-built for this study and should be regarded as a set of distinct procedural and regulatory items rather than as a validated scale; the aggregate knowledge score is accordingly treated as a descriptive summary of item-level performance rather than as a validated measure of a single underlying construct.

### 2.5. Instrument Structure

The final instrument comprised 13 scored items—10 knowledge statements and three clinical vignettes—together with one randomised vignette, an attitude section and an education section. All items were mandatory; therefore, no partially completed questionnaires were received. Each scored item combined the response and the respondent’s confidence in a single choice. For knowledge statements, the options were: True—I am confident, True—I am not confident, False—I am confident, False—I am not confident, and I do not know. The clinical vignettes used the same structure, applied to the decision options. This format was adopted to minimise respondent burden and protect the completion rate; the trade-off is that confidence was captured dichotomously rather than on a graded scale, which precludes the construction of a continuous calibration curve. Calibration in the strict sense—agreement between stated probability and observed accuracy across graded levels of confidence—cannot therefore be estimated; two coarser quantities, overall bias and confidence discrimination, are reported instead and are defined below. The knowledge items covered the legal framework of the determination, the prerequisites for the neurological examination (exclusion of neuromuscular blockade, haemodynamic adequacy, the observation interval required after anoxic injury), the apnoea test (the carbon dioxide threshold and its interpretation in chronic hypercapnia), and ancillary testing (the indication for a cerebral blood flow study and the effect of sedative drugs on electroencephalography). The three clinical vignettes addressed hypothermia, spinal reflexes, and residual sedative effect in the presence of renal failure. Before data collection, nine items (B3, B4, B5, B7, B8, B10 and the three clinical vignettes; marked with † in the item-level results (Section 3.3) were designated a priori as safety-critical according to a predefined criterion: an incorrect belief held with confidence would plausibly alter management at the bedside, either by producing an invalid determination (prerequisites, apnoea-test criteria, or acceptance of a misleading ancillary result), or by halting a valid one (spinal reflexes). The items on the legal framework (B1, B2), the inter-examination interval (B6) and the indication for ancillary testing (B9) were judged important but less likely to change bedside action directly, and were not included in the subset.

### 2.6. Randomised Vignette: Design and Allocation

Clinical vignettes are an established means of measuring clinicians’ decisions, validated against standardised patients and chart abstraction [18,19], and experimental variation in a single vignette attribute allows its influence on the decision to be isolated [20]. Following the knowledge and vignette sections, participants were presented with a clinical scenario describing a patient whose medical particulars were identical in both arms. The scenario differed between arms in a single closing sentence, describing the family as either favourably or unfavourably disposed towards organ donation. Participants were then asked whether they would initiate the brain death determination process at that point, when they would request neurological or neurosurgical consultation, and to what extent the family’s attitude had influenced their decision. Allocation was performed by simple randomisation in a 1:1 ratio, using a pseudorandom number generator executed server-side before the page was delivered to the participant. Each arm was hosted as a separate form, to which the participant was redirected after allocation. Allocation therefore depended on participants entering through the distributed study link: a participant who forwarded the address of the form to which they had been redirected would have passed on their own allocation. Participants were not informed of the existence of two versions, since disclosure would have directly compromised the behaviour under study; the consent text stated in advance that some questions might be presented in different forms to different participants. On completion, all participants were shown a debriefing screen explaining the two-arm design, stating that the patient’s medical condition had been identical in both arms, and reaffirming that brain death is determined on medical criteria alone, independently of any prospect of organ donation.

### 2.7. Outcome Measures

For each scored item, two variables were derived: accuracy (correct = 1, incorrect or “I do not know” = 0) and confidence (confident = 1, otherwise = 0). The primary outcome was the high-confidence error rate: the proportion of the 13 scored items that a participant answered incorrectly while indicating confidence. The randomised vignette was not scored for accuracy, since the two arms are compared against one another rather than against a key, and it therefore does not contribute to this denominator. Secondary outcomes were: the knowledge score (percentage of the 10 knowledge items answered correctly); the proportion of “I do not know” responses, analysed separately as an indicator of recognised uncertainty; the error rate among answers endorsed with confidence; the high-confidence error rate within the nine safety-critical items; the overconfidence bias, a measure of calibration-in-the-large, defined as the difference between the proportion of items endorsed with confidence and the proportion answered correctly; confidence discrimination (resolution), defined as the difference in accuracy between answers endorsed with confidence and answers not so endorsed; and, for the randomised vignette, the proportion of participants in each arm electing to initiate the determination process. Perceived preparedness was defined as agreement or strong agreement (4 or 5 on the five-point scale) with the statement “I would feel prepared to serve on a brain death determination board once qualified”.

### 2.8. Sample Size

The sample size was determined by the precision required for the principal descriptive proportion, namely the proportion of residents committing at least one high-confidence error. Assuming a proportion near 50% —the least favourable assumption for precision—a sample of 200 respondents yields a two-sided 95% confidence interval of approximately ±7 percentage points. This was judged adequate for the descriptive and comparative objectives of the study. The embedded randomised comparison is powered only for a large effect: with approximately 100 participants per arm, a difference of about 20 percentage points between arms (for example, 50% versus 30%) is detectable at a two-sided alpha of 0.05 with 80% power. Smaller but clinically meaningful differences would not be detected. This comparison is therefore reported as exploratory and hypothesis-generating, and a non-significant result should not be interpreted as evidence of no effect.

### 2.9. Statistical Analysis

Analyses were performed using IBM SPSS Statistics version 25.0 (IBM Corp., Armonk, NY, USA) and Python 3.12 with the statsmodels package (open-source; Python Software Foundation, Wilmington, DE, USA). Continuous variables are summarised as mean ± standard deviation or median [interquartile range] according to distribution, assessed by the Shapiro–Wilk test; categorical variables as counts and percentages, with Wilson 95% confidence intervals for the principal proportions. Internal consistency of the knowledge section was assessed using the Kuder–Richardson 20 coefficient and that of the three self-efficacy attitude items (competence to explain brain death to a family, perceived preparedness, and adequacy of training) using Cronbach’s alpha; the two remaining attitude items address distinct concerns and were not treated as a scale. Group comparisons used Student’s *t*-test or one-way analysis of variance for normally distributed data, and the Mann–Whitney U or Kruskal–Wallis test otherwise; categorical variables were compared using the chi-square or Fisher’s exact test. Trends across ordered categories were tested with the linear-by-linear association test, and associations between ordinal variables with Spearman’s coefficient. For univariable comparisons, residents in their third year or later were classified as senior. Item-level results are presented as a matrix of accuracy and high-confidence error rates. The correspondence between confidence and accuracy was examined in two ways: overall bias (calibration-in-the-large) by computing the overconfidence bias, and confidence discrimination by comparing accuracy among items endorsed with confidence against accuracy among items not so endorsed. Because each participant contributed 13 observations, this comparison was conducted at the participant × item level using logistic regression fitted by generalised estimating equations with an exchangeable working correlation structure, to account for the clustering of responses within participants [21]. Independent predictors of the knowledge score were identified by multivariable linear regression. Because the participant-level high-confidence error rate was zero in half of respondents and right-skewed, its predictors were examined instead at the participant × item level, by logistic regression fitted by generalised estimating equations with an exchangeable working correlation structure, the outcome being whether a given item was answered incorrectly with confidence; the model was repeated within confidently endorsed answers only. Both analyses used year of residency, duration of intensive care rotation, receipt of formal instruction in brain death (yes/no), number of brain death determinations attended, and institution type as covariates. Year of residency, duration of intensive care rotation and number of determinations attended were entered as ordinal terms, and institution type as university hospital versus other. The respective contributions of formal instruction and of accumulated case exposure to high-confidence error were examined within this model. Perceived preparedness was examined by multivariable logistic regression with year of residency, case exposure, formal instruction, institution type and knowledge score as covariates. Because year of residency and case exposure are collinear, the model was repeated omitting year of residency; as a sensitivity analysis, the five-point preparedness score was analysed by linear regression with the full covariate set. For the randomised vignette, the proportion electing to initiate the determination process was compared between arms using the chi-square test, with an effect estimate adjusted for year of residency, case experience, formal instruction and institution type obtained by multivariable logistic regression. As a post hoc check on the integrity of allocation, responses were inspected in order of receipt, and the randomised comparison was repeated after excluding a run of responses incompatible with 1:1 allocation. Further sensitivity analyses repeated the main results after excluding the inter-examination interval item, whose keyed answer is specific to national regulation, and after excluding respondents aged 45 years or older. A two-sided *p*-value below 0.05 was considered statistically significant. The primary and secondary outcomes, the item-level analysis, the univariable comparisons of the knowledge score by formal instruction and seniority, the GEE comparison of accuracy by confidence, the multivariable models for the knowledge score, high-confidence error and perceived preparedness, and the between-arm comparison of the randomised vignette were specified before data collection. The following analyses were post hoc and are identified as such where reported: the inspection of allocation integrity and the exclusion of the 20 non-randomised responses, the re-estimation of the knowledge-score model without these responses, the preparedness model omitting year of residency, the sensitivity analyses excluding the inter-examination interval item and respondents aged 45 years or older, and the univariable comparison of high-confidence error by seniority. These post hoc analyses should be regarded as exploratory.

## 3. Results

### 3.1. Participants

The survey link was distributed between 31 August and 20 September 2026. A total of 207 forms were submitted; the platform does not record how many residents opened the link without submitting. One respondent declined consent at the opening screen, so that the form was submitted without further responses; this record was excluded, leaving 206 completed responses for analysis (Table 1) (randomised arm A, *n* = 111; arm B, *n* = 95). No partially completed questionnaires were received, since all items were mandatory. The mean age of respondents was 32.1 ± 5.8 years (median 31, IQR 28–34). Of the respondents, 105 (51.0%) were male, 97 (47.1%) were female, and 4 (1.9%) preferred not to state. Residents in their fourth and fifth (or later) years together made up just over half of the sample (51.5%). Most respondents worked in training and research hospitals (130, 63.1%), followed by university hospitals (59, 28.6%). Exposure to the clinical setting was substantial but uneven. In all, 124 residents (60.2%) had completed more than six months of intensive care rotation, whereas 23 (11.2%) had never rotated through an intensive care unit. Thirty-nine residents (18.9%) had never participated in a brain death determination, and the same number (18.9%) had never been involved in an apnoea test. Only 80 participants (38.8%) reported having received any formal instruction on brain death, and almost half (98, 47.6%) did not know whether their institution had a written brain death protocol. When asked where their knowledge had principally come from, residents cited residency lectures (71, 34.5%) and observation of senior physicians (69, 33.5%) in almost equal measure, while 27 (13.1%) reported having had no formal source at all.

**Table 1:** Participant characteristics (*n* = 206).

Characteristic	*n* (%)
Age, years—mean ± SD	32.1 ± 5.8
Age, years—median (IQR)	31 (28–34)
Sex	
Male	105 (51.0)
Female	97 (47.1)
Prefer not to state	4 (1.9)
Year of residency	
First	31 (15.0)
Second	33 (16.0)
Third	36 (17.5)
Fourth	51 (24.8)
Fifth or later	55 (26.7)
Institution type	
Training and research hospital	130 (63.1)
University hospital	59 (28.6)
Other	17 (8.3)
Intensive care rotation	
None	23 (11.2)
Less than 3 months	22 (10.7)
3–6 months	37 (18.0)
More than 6 months	124 (60.2)
Brain death determinations attended	
None	39 (18.9)
1–2	69 (33.5)
3–5	59 (28.6)
More than 5	39 (18.9)
Apnoea tests performed or attended	
None	39 (18.9)
1–2	78 (37.9)
3–5	51 (24.8)
More than 5	38 (18.4)
Formal instruction on brain death	
Yes	80 (38.8)
No	126 (61.2)
Written institutional protocol	
Yes	61 (29.6)
No	47 (22.8)
Do not know	98 (47.6)
Principal source of knowledge	
Residency lectures	71 (34.5)
Observation of senior physicians	69 (33.5)
Congress or course	6 (2.9)
Own reading	33 (16.0)
No formal source	27 (13.1)

### 3.2. Knowledge, Confidence and Their Correspondence

Across the 13 scored items, residents answered 80.9% of items correctly and endorsed 64.2% of their answers with confidence. The mean knowledge score, calculated over the 10 knowledge statements, was 83.0 ± 16.7% (median 90, IQR 80–90). Residents therefore claimed confidence less often than their accuracy warranted: the mean overconfidence bias was −16.7 ± 22.7 percentage points, indicating systematic under-confidence rather than overconfidence. Responses of “I do not know” were infrequent, accounting for 4.4% of all answers (mean 0.57 items per respondent). Confidence discriminated well between correct and incorrect answers. Items endorsed with confidence were answered correctly in 90.8% of instances (*n* = 1719), compared with 63.3% of items not so endorsed (*n* = 959). In the generalised estimating equation model, a confident answer had 5.55 times the odds of being correct (95% CI 4.44–6.94; *p* < 0.001); conversely, 9.2% of confidently endorsed answers were wrong. Nevertheless, at the level of the individual respondent, high-confidence errors were not rare. The mean high-confidence error rate was 5.9 ± 7.3% of items (median 7.7, IQR 0–7.7), and the corresponding rate within the nine safety-critical items was 6.3 ± 9.5%. In total, 104 residents (50.5%, 95% CI 43.7–57.2%) committed at least one high-confidence error, and 12 (5.8%) committed three or more. When the inter-examination interval item was excluded, the mean high-confidence error rate was 5.4 ± 7.5% and 90 residents (43.7%) committed at least one such error.

### 3.3. Item-Level Performance

High-confidence errors were not distributed evenly across the instrument but concentrated in a small number of domains (Table 2 and Figure 1). The highest rate was observed for the interpretation of spinal reflexes: 14.6% (95% CI 10.4–20.0%) of residents stated with confidence that a flexion movement elicited by a painful stimulus should halt the determination process, and only 67.0% answered this item correctly. The required interval between the two neurological examinations was the second-most frequent source of high-confidence error (12.6%, 95% CI 8.8–17.9%; 64.6% correct) and was also the item most often answered “I do not know” (12.1%). The hypothermia vignette (9.7%, 95% CI 6.4–14.5%; 71.8% correct) and the interpretation of the apnoea test in chronic hypercapnia (9.2%, 95% CI 6.0–14.0%; 72.8% correct) followed. At the opposite end, three items were answered correctly by more than 90% of respondents—the independence of the determination from donor status (92.7%), the carbon dioxide threshold of the apnoea test (91.7%), and the influence of sedative drugs on electroencephalography (98.5%)—with high-confidence error rates of 1.9%, 1.5%, and 0% respectively.

**Figure 1:**
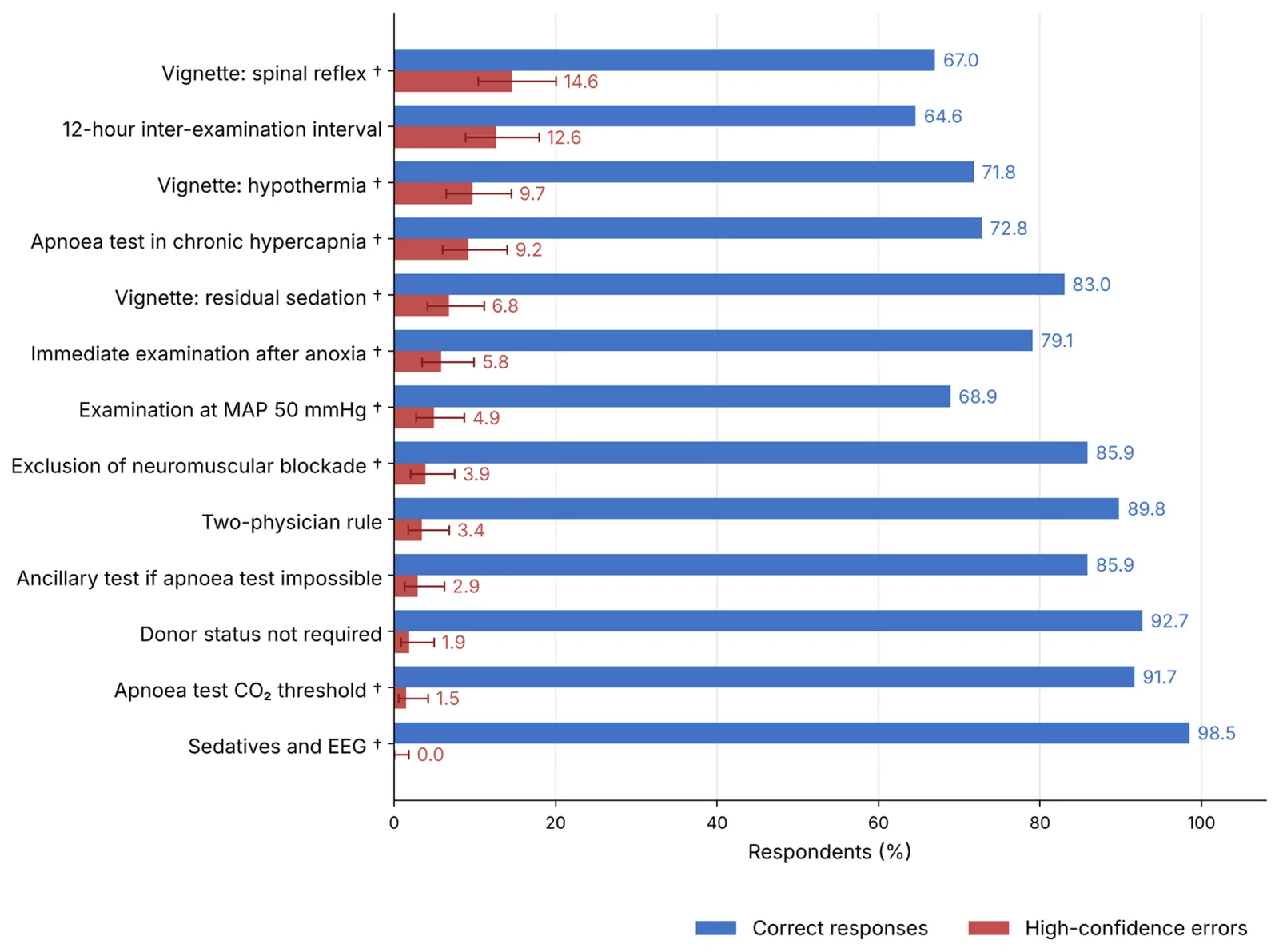
Item-level accuracy and high-confidence errors (*n* = 206). Blue bars show the proportion of respondents answering each item correctly; red bars show the proportion committing a high-confidence error, defined as an incorrect response endorsed with confidence; error bars show Wilson 95% confidence intervals for the high-confidence error rate. Items are ordered by high-confidence error rate. † denotes membership of the a priori safety-critical subset. MAP, mean arterial pressure; EEG, electroencephalography.

**Table 2:** Item-level accuracy, confidence and high-confidence error.

Item	Content	Correct (%)	Confident (%)	High-Confidence Error, % (95% CI)	“Do Not Know” (%)
V2 †	Vignette 2—flexion movement to painful stimulus: would you continue the determination?	67.0	46.6	**14.6 (10.4–20.0)**	0.0
B6	At least 12 h must elapse between the two neurological examinations (True)	64.6	41.3	**12.6 (8.8–17.9)**	12.1
V1 †	Vignette 1—hypothermia (oesophageal temperature 31.2 °C): would you proceed to apnoea testing?	71.8	68.0	**9.7 (6.4–14.5)**	0.0
B8 †	In chronic hypercapnia the apnoea test may be interpreted using the standard threshold (False)	72.8	60.7	**9.2 (6.0–14.0)**	7.8
V3 †	Vignette 3—midazolam infusion with renal failure: would you begin the examination?	83.0	65.5	**6.8 (4.1–11.1)**	0.0
B5 †	Examination may begin immediately after anoxic brain injury (False)	79.1	62.1	**5.8 (3.4–9.9)**	5.3
B4 †	Brainstem examination is valid at a mean arterial pressure of 50 mmHg (False)	68.9	51.9	**4.9 (2.7–8.7)**	10.2
B3 †	Neuromuscular blockade must be excluded; confirm with train-of-four if in doubt (True)	85.9	61.7	**3.9 (2.0–7.5)**	5.3
B2	Determination is made by two physicians as specified in current legislation (True)	89.8	71.8	**3.4 (1.7–6.8)**	1.9
B9	If apnoea testing is impossible, a cerebral blood flow study may complete the determination (True)	85.9	62.1	**2.9 (1.3–6.2)**	5.3
B1	Determination requires the patient to be an organ donor (False)	92.7	87.9	**1.9 (0.8–4.9)**	2.9
B7 †	Apnoea test requires PaCO_2_ ≥60 mmHg or a rise of ≥20 mmHg from baseline (True)	91.7	72.8	**1.5 (0.5–4.2)**	5.3
B10 †	Electroencephalography may be misleading in the presence of high-dose sedatives (True)	98.5	82.0	**0.0 (0.0–1.8)**	1.0

† Item belonging to the a priori safety-critical subset. Values in the high-confidence error column (the primary outcome) are shown in bold. CI, Wilson confidence interval; *n* = 206 for all items.

### 3.4. Predictors of Knowledge and of High-Confidence Error

Residents who had received formal instruction scored substantially higher on the knowledge items than those who had not (88.6 ± 10.4% versus 79.4 ± 18.9%; *p* < 0.001), as did senior residents (third year or later, *n* = 142) compared with first- and second-year residents (*n* = 64; 86.4 ± 13.3% versus 75.5 ± 20.6%; *p* < 0.001). In multivariable linear regression, the knowledge score was independently associated with formal instruction (β = 4.92, *p* = 0.034), with the number of brain death determinations attended (β = 2.96 per category, *p* = 0.033) and, inversely, with working in a university hospital (β = −5.51, *p* = 0.023). The model explained 21.5% of the variance (*p* < 0.001). The association with institution type was, however, attributable to a single cluster of 20 responses (see Randomised vignette). When these were excluded, the coefficient for university hospital was −3.62 (*p* = 0.153), and the mean knowledge score of the remaining university-hospital residents (82.8 ± 17.2%, *n* = 39) did not differ from that of residents elsewhere (85.1 ± 14.1%, *n* = 147; *p* = 0.479); case exposure remained associated with the knowledge score (β = 3.23, *p* = 0.017), whereas formal instruction did not (β = 3.07, *p* = 0.170); the association between formal instruction and the knowledge score is therefore not robust to this post hoc exclusion. The high-confidence error rate behaved differently. It did not differ according to formal instruction (6.0 ± 7.2% versus 5.9 ± 7.3%; *p* = 0.936), and in a post hoc univariable comparison was in fact slightly higher among senior residents (6.5 ± 7.0% versus 4.7 ± 7.7%; median 7.7 [IQR 0–7.7] versus 0 [IQR 0–7.7]; Mann–Whitney *p* = 0.015). This reflects the greater frequency with which senior residents declared confidence (72.9 ± 22.4% versus 44.8 ± 32.0% of items; *p* < 0.001) rather than poorer judgement of their own answers: among confidently endorsed answers, the error rate was 8.9% in senior and 10.5% in junior residents. In the item-level clustered logistic model, no covariate was associated with high-confidence error: year of residency (odds ratio 0.97, 95% CI 0.75–1.23), duration of intensive care rotation (1.22, 0.87–1.72), formal instruction (0.92, 0.62–1.37), case exposure (1.07, 0.86–1.34) and university hospital (1.27, 0.85–1.89; all *p* ≥ 0.25). The result was the same when the model was restricted to confidently endorsed answers (all *p* ≥ 0.14). Neither seniority, intensive care exposure, formal instruction, case experience, nor institution type predicted who would make a high-confidence error.

### 3.5. Randomised Vignette

In the randomised scenario, the proportion of residents electing to initiate the brain death determination process was 85.6% (95/111; 95% CI 77.9–90.9%) in arm A, in which the family was described as favourably disposed toward organ donation, and 87.4% (83/95; 95% CI 79.2–92.6%) in arm B, in which the family was described as opposed (difference −1.8 percentage points; χ^2^ *p* = 0.866). After adjustment for seniority, case experience, formal instruction and institution type, the odds ratio for arm B relative to arm A was 1.29 (95% CI 0.54–3.05; *p* = 0.568). The timing of requested neurological consultation did not differ significantly between arms: immediate consultation was chosen by 71.2% of arm A and 77.9% of arm B, consultation after 24 h by 22.5% and 12.6%, and no consultation by 6.3% and 9.5% respectively (*p* = 0.153). Self-reported influence, however, diverged sharply from behaviour. Residents in arm A rated the family’s attitude as more influential on their decision than those in arm B (2.50 ± 1.55 versus 1.61 ± 1.14 on a five-point scale; *p* < 0.001), and the proportion stating that the family’s attitude had not influenced them at all was 42.3% in arm A compared with 70.5% in arm B (rating of 4 or 5: 29 [26.1%] versus 8 [8.4%]). The decisions themselves nonetheless did not differ detectably between the two arms. Baseline characteristics of the two arms are presented in Table 3. The unequal arm sizes (111 versus 95) are consistent with simple, unblocked randomisation, under which a split at least this uneven is expected in about 30% of allocations of 206 participants (binomial *p* = 0.30); allocation was not blocked, so equal arm sizes were not guaranteed by design. The arms were comparable in age, sex, seniority, intensive care exposure, case experience and formal instruction, but differed in institution type (*p* = 0.006). A post hoc inspection of responses in order of receipt showed that this imbalance arose from a single episode: on 18 September, between 14:32 and 18:38, all 20 responses received from university-hospital residents were in arm A, a pattern incompatible with 1:1 allocation (probability below one in a million) and consistent with onward sharing, within one department, of the address of the arm A form rather than of the study link. These 20 respondents (13 in their first or second year; 14 had never attended a determination) were retained in the descriptive analyses but cannot be regarded as randomised. When they were excluded, the arms were balanced in institution type (*p* = 0.501) and the result was materially unchanged (arms of 91 and 95): 85.7% (78/91) in arm A and 87.4% (83/95) in arm B elected to initiate determination (*p* = 0.908; adjusted odds ratio 1.29, 95% CI 0.53–3.12; *p* = 0.577).

### 3.6. Attitudes, Perceived Preparedness and Barriers

Agreement with the attitude statements was low throughout (Table 4). Only 18.0% (37/206; 95% CI 13.3–23.8%) of residents agreed or strongly agreed that they would feel prepared to serve on a brain death determination board once qualified (mean 2.39 ± 1.26). Similar proportions considered the instruction they had received during residency adequate (26.2%; mean 2.63 ± 1.26) and felt competent to explain the concept of brain death to a family (27.2%; mean 2.73 ± 1.30). Concern about the legal consequences of the determination was endorsed by 24.3%, and discomfort at the intertwining of determination and donation by 20.4%. Perceived preparedness varied by seniority (9.7% of first-year, 9.1% of second-year and 5.6% of third-year residents, compared with 25.5% of fourth-year and 29.1% of fifth-year or later residents), with formal instruction (27.5%, 95% CI 18.9–38.1%, versus 11.9%, 95% CI 7.3–18.7%; *p* = 0.008) and with case experience (5.1%, 95% CI 1.4–16.9%, among those who had attended no determinations, rising to 33.3%, 95% CI 20.6–49.0%, among those who had attended more than five; *p* for trend <0.001). Perceived preparedness was not associated with the knowledge score (84.9 ± 12.8% among residents who felt prepared versus 82.6 ± 17.4% among those who did not; *p* = 0.860). In multivariable logistic regression including year of residency, case exposure, formal instruction, institution type and knowledge score, the knowledge score carried an odds ratio of 0.985 per percentage point (*p* = 0.270), and no covariate reached statistical significance; year of residency and case exposure were, however, strongly correlated (Spearman ρ = 0.59). In a post hoc model omitting year of residency, case exposure was independently associated with preparedness (odds ratio 1.72 per category, 95% CI 1.13–2.64; *p* = 0.012), formal instruction less clearly so (odds ratio 2.08, 95% CI 0.93–4.64; *p* = 0.075), and the knowledge score not at all (*p* = 0.445). In the sensitivity analysis treating preparedness as a five-point score with the full covariate set, both case exposure (β = 0.29 per category, *p* = 0.008) and formal instruction (β = 0.39, *p* = 0.041) were independently associated, whereas year of residency (*p* = 0.807) and knowledge score (*p* = 0.301) were not. The preparedness score correlated with the proportion of items a resident endorsed with confidence (ρ = 0.29, *p* < 0.001) but not with the proportion answered correctly (ρ = 0.03, *p* = 0.706). The most frequently reported barriers (Table 5) were difficulty communicating with families (146, 70.9%), concern about legal consequences (133, 64.6%), anxiety regarding forensic proceedings (122, 59.2%), and inadequate training (110, 53.4%). The absence of a written institutional protocol was cited by 83 residents (40.3%). Only 17 respondents (8.3%) reported perceiving no barriers at all. The contrast between the 24.3% who agreed with the personal statement of legal concern and the 64.6% who selected legal consequences from the list of barriers reflects the framing of the two questions: the former asks about the respondent’s own present concern, the latter about obstacles encountered or anticipated in the determination process in general.

**Table 4:** Attitudes and perceived barriers (*n* = 206).

Statement	Mean ± SD	Agree or Strongly Agree, *n* (%; 95% CI)
I am concerned about the legal consequences of determining brain death	2.53 ± 1.31	50 (24.3; 18.9–30.6)
I feel competent to explain the concept of brain death to a family	2.73 ± 1.30	56 (27.2; 21.6–33.6)
I am uncomfortable that determination and organ donation are intertwined	2.50 ± 1.31	42 (20.4; 15.5–26.4)
I would feel prepared to serve on a brain death determination board once qualified	2.39 ± 1.26	37 (18.0; 13.3–23.8)
I consider the instruction I received during residency adequate	2.63 ± 1.26	54 (26.2; 20.7–32.6)

CI, Wilson 95% confidence interval. Means are on a five-point scale from 1 (strongly disagree) to 5 (strongly agree); no inferential tests are reported in this table.

**Table 5:** Perceived barriers (more than one response permitted).

Barrier	*n* (%)
Difficulty communicating with families	146 (70.9)
Concern about legal consequences	133 (64.6)
Anxiety regarding forensic proceedings	122 (59.2)
Inadequate training	110 (53.4)
Absence of a written institutional protocol	83 (40.3)
Time and workload	68 (33.0)
Lack of supervision	54 (26.2)
No barriers perceived	17 (8.3)

### 3.7. Instrument Reliability

The Kuder–Richardson 20 coefficient was 0.594 for the 10 knowledge items and 0.564 across all 13 scored items; Cronbach’s alpha for the three self-efficacy items was 0.861; across all five attitude items, which do not form a single scale, it was 0.597.

## 4. Discussion

In this web-based survey of a national convenience sample of 206 anaesthesiology and reanimation residents, item-level knowledge of the criteria and prerequisites for brain death determination was generally sound, and residents’ confidence was, on the whole, well matched to their accuracy: answers endorsed with confidence were correct in more than nine cases out of ten, and residents claimed confidence less often than their accuracy would have justified. Three findings nonetheless qualify this reassuring picture.

### 4.1. Confident Error Is Uncommon Overall but Concentrated in Specific Domains

Half of the residents committed at least one high-confidence error, and these errors concentrated in four content areas. The most frequent concerned the interpretation of spinal reflexes. Spinally mediated movements in brain-dead patients are well described and do not invalidate the diagnosis [1,22,23,24], yet the belief that they do carries a specific and asymmetric cost: it does not produce a false determination of death, but it delays or abandons a correct one, prolonging futile organ support and the family’s uncertainty. The second was the interval required between the two neurological examinations—an item derived directly from national regulation, and the item most often answered “I do not know”. This item nonetheless requires a caveat. The 12 h interval is the requirement of the national regulation [3], but national guidance permits the waiting period to be dispensed with when an ancillary test demonstrates absent cerebral blood flow [25], and current international guidance considers a single examination sufficient in adults [1,2,26]. Some residents who confidently rejected the statement may therefore have been reasoning from these sources rather than from a misconception, and the high-confidence error rate for this item should be read as an upper bound; excluding it left the overall picture unchanged. The remaining two items—the invalidity of the neurological examination in hypothermia and the interpretation of the apnoea test in chronic hypercapnia—are both prerequisites rather than findings, and both are addressed explicitly in current consensus guidance [1,2,27]. Errors of this kind are consequential precisely because they arise before the examination begins: a determination founded on an invalid prerequisite is invalid in its entirety, regardless of how carefully the subsequent steps are performed. These four domains constitute a concrete and narrow target for instruction: not diffuse knowledge deficits requiring wholesale curricular revision, but specific misconceptions that a short, focused intervention could plausibly address. Whether such an intervention reduces high-confidence error was not tested here.

### 4.2. Preparedness Is Low and Is Not Explained by Knowledge

The most striking finding of this study is the dissociation between item-level knowledge and perceived preparedness. Although the mean knowledge score exceeded 80%, fewer than one resident in five felt prepared to serve on a brain death determination board upon qualification—a role that, under Law No. 2238, a substantial proportion of them will occupy. Critically, perceived preparedness was not associated with the knowledge score. Preparedness rose instead with seniority, with formal instruction and, most steeply, with the number of determinations actually attended. Two interpretive limits must be stated at once. First, perceived preparedness is a self-reported judgement: it is not a measure of clinical competence, and nothing in this study establishes how residents would actually perform at the bedside. Second, the association between case exposure and preparedness is cross-sectional: seniority and exposure were strongly correlated, exposure was not assigned, and residents who feel more prepared may also seek out more determinations. The data therefore show that the sense of unpreparedness tracks experience rather than knowledge; they do not show that increasing exposure would cause preparedness, still less competence, to improve. With these limits, the pattern argues against the conclusion that would ordinarily be drawn from a knowledge survey: the deficit identified here appears to be less one of information than of experience and supervised exposure. Nearly one in five residents had never participated in a determination at all, and a similar proportion had never been involved in an apnoea test. If this interpretation is correct, additional didactic content alone would be unlikely to close the gap. Structured bedside supervision and simulation-based practice, with explicit attention to the four error-prone domains identified above, are the plausible complements; formal instruction, which only 39% of residents had received and which was itself associated with greater preparedness, remains necessary. Two further qualifications apply. At the group level, knowledge and preparedness both rose with seniority and case exposure (Figure 2); the dissociation we describe holds between individuals, not between strata. The knowledge score was also subject to a ceiling effect and to modest internal consistency, both of which attenuate correlations, so the absence of an association with preparedness may be partly a property of the measure. The association between formal instruction and the knowledge score should likewise be read cautiously: it was present in the primary model but not after the post hoc exclusion of the 20 non-randomised responses, whereas the association with case exposure persisted. That preparedness tracked declared confidence rather than accuracy nonetheless points in the same direction: what residents report lacking is less knowledge than the experience that converts knowledge into assurance. This is consistent with self-efficacy theory, in which direct mastery experience is the most powerful source of perceived capability [28], and with evidence that expert performance is acquired through supervised, deliberate practice rather than through the accumulation of knowledge alone [29]. The finding also accords with calls for standardised, competency-oriented education in brain death determination, which have emphasised that exposure during training is inconsistent and that written knowledge does not translate directly into bedside performance [30,31]. Simulation-based training in particular has been shown elsewhere to improve trainees’ competence both in conducting the determination and in discussing it with families [32,33]; the present study did not evaluate any such intervention. The barriers residents reported are consistent with this interpretation. Difficulty communicating with families was the most frequently cited obstacle, endorsed by more than 70% of residents, while only around a quarter felt competent to conduct such a conversation. Communication with families is a teachable skill that is rarely taught explicitly in this context, and the agreement between these two findings identifies it as a specific and actionable target. How the family is approached and informed is, moreover, associated with its subsequent decision about donation [34]. Institutional factors also emerged. Nearly half of respondents did not know whether their own institution possessed a written brain death protocol, and 40% cited the absence of such a protocol as a barrier. Where a legally defined procedure is carried out without a locally documented pathway, uncertainty is a rational response rather than a personal deficiency.

### 4.3. The Decision to Initiate Determination Did Not Differ by Donation Context

In the embedded randomised vignette, residents presented with a medically identical patient elected to initiate the determination process at similar rates whether the family had been described as favourably or unfavourably disposed toward organ donation. The difference between arms was under two percentage points and remained non-significant after adjustment. Taken at face value this is reassuring: international guidance and national regulation alike require that determination proceed on medical grounds alone and independently of any prospect of donation, and at the level of trainee decision-making no large departure from that separation was detected. The comparison must nonetheless be regarded as exploratory, for three reasons. First, the comparison was subject to a marked ceiling effect: approximately 86% of residents in both arms elected to proceed, leaving little room in which a difference could have manifested. Second, the study was powered only to detect a difference of roughly 20 percentage points; a smaller but still meaningful effect would not have been detected. The absence of a statistically significant difference should therefore not be read as demonstrating the absence of any effect. Third, one run of 20 responses showed clear evidence that the address of a single arm had been shared, and those respondents were not randomised. Their exclusion did not alter the result, but because this breach was detected only through inspection of response timing, less conspicuous breaches cannot be excluded and allocation integrity cannot be guaranteed for the remaining responses. The episode illustrates a vulnerability of link-based allocation in anonymous web surveys, which future studies should design against, for example with single-use links or individual tokens. The appropriate conclusion is therefore not that the family’s attitude has no influence on the decision to initiate determination, but that no large effect was detected in a comparison with limited power and imperfectly protected allocation. A secondary observation merits comment. Although behaviour did not differ between arms, self-reported influence did: residents in the favourable-family arm rated the family’s attitude as considerably more influential on their decision, and more than half of them reported some degree of influence, which tempers the reassurance offered by the behavioural result. Whether this reflects heightened awareness of donation as a consideration when it is salient and positive, or a difference in social desirability between the two framings, cannot be determined from these data. The observation is exploratory and is offered as a hypothesis for further work.

### 4.4. Relation to Previous Work

Previous surveys of physicians’ knowledge of brain death have reported substantial deficiencies, and have not consistently found experience to be protective [4,5]. Our findings differ in an instructive way: the residents surveyed here performed well on knowledge items, and the shortfall we identified lies elsewhere—in confidence, in exposure and in institutional support. This divergence may reflect genuine differences between settings, but it may equally reflect what is being measured: a survey that reports only the proportion of correct answers cannot distinguish a resident who knows the criteria and feels ready from one who knows them and does not. The present study suggests that this distinction is not incidental. The lower knowledge score observed among university-hospital residents should not be interpreted as a property of that setting. It was driven by a single cluster of 20 responses, apparently from one department and consisting largely of junior residents who had never attended a determination; without them, the difference disappeared. Because the institution was not recorded, clustering of responses by centre could not be modelled, and comparisons between institution types in this dataset are unreliable.

### 4.5. Educational Implications and Priorities for Future Research

The educational measures suggested by these findings—supervised bedside exposure, targeted correction of the four misconceptions identified, explicit training in communication with families and the availability of a written local protocol—are implications drawn from cross-sectional associations and from residents’ own reports; they are not effects demonstrated by this study. None was evaluated as an intervention, the study did not test whether targeted training reduces high-confidence error, and it did not test whether increased exposure improves preparedness or bedside performance. They are offered as priorities for future work: a before-and-after or cluster-randomised evaluation of a short, focused module addressing the four error-prone domains, with high-confidence error as the outcome; assessment of competence by direct observation or simulation rather than by self-report; and the use of single-use survey links or individual tokens to protect allocation in future embedded experiments.

### 4.6. Limitations

This study has several limitations. It is cross-sectional and based on self-report, and can therefore establish associations but not causal relationships; in particular, confidence and perceived preparedness are self-reports that were not validated against observed performance, and neither should be equated with clinical competence. Distribution relied on professional networks of residents and on departmental chairs. Because the number of residents reached could not be established, a conventional response rate cannot be reported; the sample is a national convenience sample, it is not representative of anaesthesiology and reanimation residents in Türkiye, and both selection bias and non-response bias are plausible. For the same reason, and because the name of the institution was not recorded, neither the number of participating centres nor the clustering of responses within centres could be established. Multiple submissions could not be technically prevented, although no identical records were found. Residents with a particular interest in the subject may have been more likely to participate, which would tend to overestimate knowledge; the findings on preparedness would, if anything, be conservative under this assumption. The anonymous design, adopted deliberately to protect participants, precluded verification of eligibility. A small number of respondents reported ages atypical for residency training, and a sensitivity analysis excluding the nine respondents aged 45 years or older (*n* = 197) gave an overall accuracy of 80.7%, a mean high-confidence error rate of 5.9 ± 7.2% and perceived preparedness of 16.8%, which do not differ materially from the results reported. Confidence was captured dichotomously rather than on a graded scale, in order to limit respondent burden and protect the completion rate. This precludes the construction of a continuous calibration curve or the calculation of a Brier score, and confines the analysis to overall bias and to the discrimination between confident and non-confident responses, rather than calibration in the strict sense. The internal consistency of the knowledge section was below the conventional threshold. The instrument was purpose-built for this study and was pretested with specialists for clarity and content, but was not subjected to formal psychometric validation. It was designed to sample a set of distinct procedural and regulatory requirements rather than to measure a single latent trait, a situation in which internal consistency coefficients are expected to be modest [35]. Nevertheless, this limits the interpretation of the aggregate knowledge score, which should be regarded as a descriptive summary, and the results should be read primarily at the item level. The primary outcome (high-confidence error) and the item-level results do not depend on the scale properties of the aggregate score. Knowledge items were presented in true-false format, which carries a 50% baseline probability of a correct answer by chance. The inclusion of an explicit “I do not know” option mitigates but does not eliminate this, and the relatively high overall knowledge score should be interpreted with this inflation in mind. The survey platform did not permit navigation between sections to be disabled, so the possibility that some respondents revised earlier answers cannot be excluded. Allocation to the randomised vignette was compromised for 20 respondents, as described above; they were identified only post hoc, less conspicuous breaches cannot be excluded, and the comparison was powered only for a large effect and was subject to a ceiling effect, so it should be regarded as exploratory and cannot establish the absence of a modest effect. The unequal arm sizes reflect simple, unblocked randomisation rather than a further breach. The answer key followed national regulation, which differs from international guidance on the inter-examination interval and on the carbon dioxide criterion of the apnoea test. Finally, the study was conducted within a single national regulatory framework, and findings concerning regulatory knowledge in particular may not generalise to other jurisdictions.

## 5. Conclusions

Anaesthesiology and reanimation residents in this national convenience sample answered most knowledge items correctly and were, if anything, insufficiently confident in what they knew. In an exploratory randomised vignette, the decision to initiate the determination process did not differ according to the family’s attitude toward organ donation; the comparison was powered only to detect a large effect, however, and allocation was compromised for a subset of responses, so the absence of a modest effect cannot be inferred. Fewer than one in five felt prepared to assume a responsibility that national law will shortly place upon many of them. In univariable and sensitivity analyses, this self-reported sense of unpreparedness was associated not with how much they knew but with how many determinations they had attended and whether they had received formal instruction; it is not a measure of clinical competence, and the associations are cross-sectional. High-confidence errors, though uncommon overall, clustered in four identifiable domains: the interpretation of spinal reflexes, the interval between examinations, the invalidity of examination in hypothermia, and the apnoea test in chronic hypercapnia. These findings identify supervised clinical exposure, targeted correction of specific misconceptions, explicit training in communication with families and the local availability of a written determination protocol as educational priorities, whose effect on preparedness and on bedside performance remains to be tested in intervention studies.

## Figures and Tables

**Figure 2:**
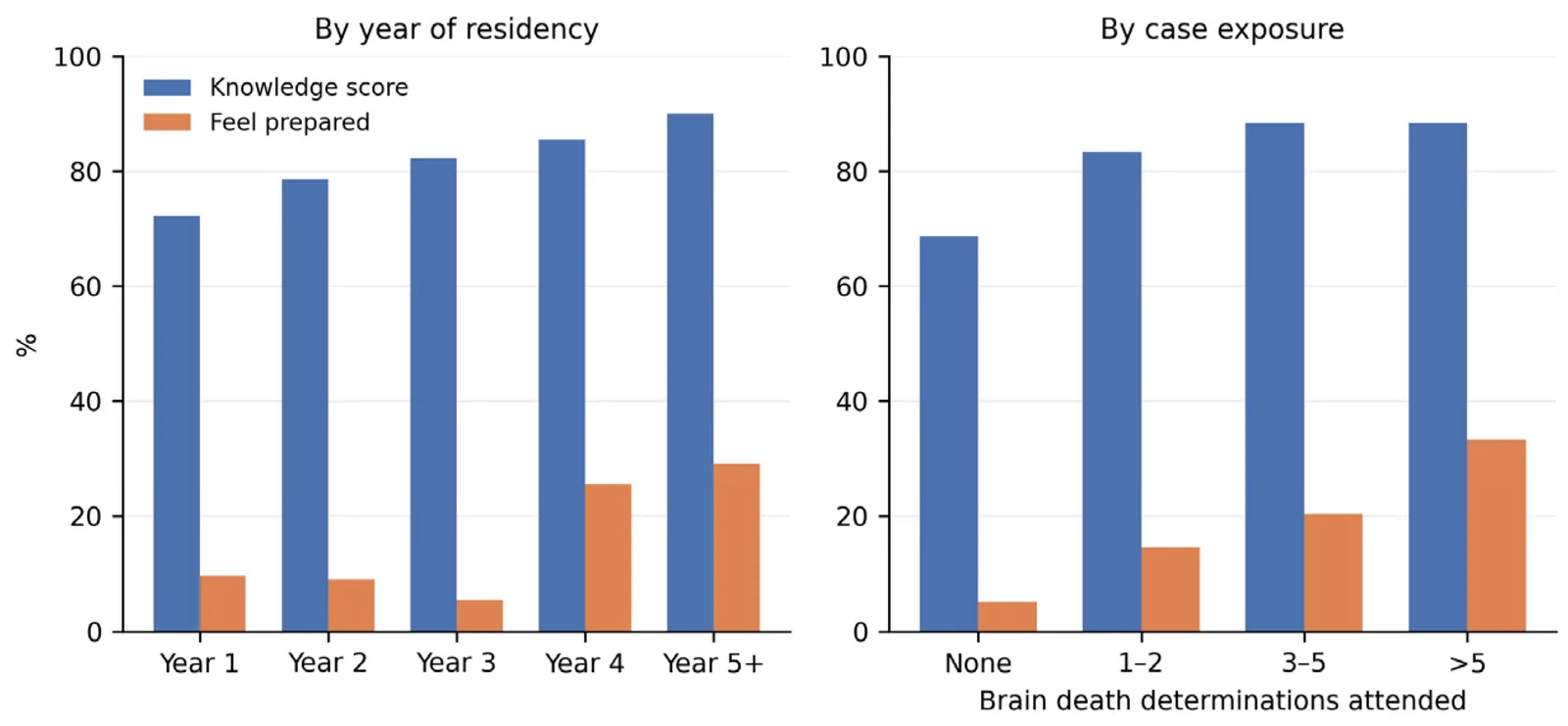
Knowledge score and perceived preparedness, by year of residency (**left**) and by number of brain death determinations attended (**right**). Blue bars show the mean knowledge score; orange bars show the proportion of residents agreeing or strongly agreeing that they would feel prepared to serve on a brain death determination board once qualified. Both rise with seniority and case exposure, but perceived preparedness remains low in every stratum, including those in which the mean knowledge score approaches 90%.

**Table 3:** Baseline characteristics by randomised arm.

Characteristic	Arm A (*n* = 111)	Arm B (*n* = 95)	*p*-Value
Age, years—mean ± SD	32.6 ± 6.2	31.4 ± 5.2	0.093
Sex			0.693
Male	56 (50.5)	49 (51.6)	
Female	52 (46.8)	45 (47.4)	
Prefer not to state	3 (2.7)	1 (1.1)	
Year of residency			0.885
First	16 (14.4)	15 (15.8)	
Second	17 (15.3)	16 (16.8)	
Third	19 (17.1)	17 (17.9)	
Fourth	26 (23.4)	25 (26.3)	
Fifth or later	33 (29.7)	22 (23.2)	
Institution type			0.006
Training and research hospital	60 (54.1)	70 (73.7)	
University hospital	42 (37.8)	17 (17.9)	
Other	9 (8.1)	8 (8.4)	
Intensive care rotation			0.431
None	16 (14.4)	7 (7.4)	
Less than 3 months	12 (10.8)	10 (10.5)	
3–6 months	20 (18.0)	17 (17.9)	
More than 6 months	63 (56.8)	61 (64.2)	
Determinations attended			0.824
None	23 (20.7)	16 (16.8)	
1–2	38 (34.2)	31 (32.6)	
3–5	31 (27.9)	28 (29.5)	
More than 5	19 (17.1)	20 (21.1)	
Formal instruction			0.455
Yes	40 (36.0)	40 (42.1)	
No	71 (64.0)	55 (57.9)	

*p*-values from Student’s *t*-test (age) or the chi-square or Fisher’s exact test (categorical variables); a two-sided *p*-value < 0.05 was considered statistically significant. The imbalance in institution type arose from 20 responses received in arm A from university-hospital residents on a single afternoon (see text); in a post hoc comparison after their exclusion, *p* = 0.501.

## Data Availability

The full questionnaire is provided as Supplementary File S1. The anonymised dataset generated and analysed during the present study is available from the corresponding author on reasonable request.

## Supplementary Materials

The following supporting information can be downloaded at https://www.mdpi.com/article/10.3390/healthcare14203495/s1. File S1: Survey instrument (full questionnaire, including both versions of the randomised vignette and the debriefing text; Turkish original with English translation).

## Abbreviations

CHERRIES: Checklist for Reporting Results of Internet E-Surveys; CI: confidence interval; EEG: electroencephalography; IQR: interquartile range; MAP: mean arterial pressure; PaCO_2_: arterial partial pressure of carbon dioxide; SD: standard deviation; STROBE: Strengthening the Reporting of Observational Studies in Epidemiology.
