# Supplementary material for: Knowledge, Confidence and Preparedness for Brain Death Determination Among Anaesthesiology and Reanimation Residents: A National Web-Based Survey with an Embedded Randomised Vignette
Source: Healthcare (Basel). 2026 Oct 17;14(20):3495. doi: 10.3390/healthcare14203495 (PMC13704572; doi:10.3390/healthcare14203495)
Supplement: Supplementary file 1 [file healthcare-14-03495-s001.zip › healthcare-4632066-supplementary.pdf]

Supplementary File S1. Survey instrument

Knowledge, Confidence and Preparedness for Brain Death Determination Among Anaesthesiology and Reanimation Residents: A National Web-Based Survey With an Embedded Randomised Vignette.
Yanatma S, Kaya S.

The survey was administered in Turkish as two Google Forms (version A and version B), identical except for the closing sentence of the randomised scenario (Section 5). Part I is an English translation prepared for this submission; Part II is the Turkish original as seen by participants. All questions were mandatory. The keyed answers and the † symbol (a priori safety-critical item) are shown here for the reader and were not visible to participants. The answer key follows the national regulation (Regulation on Organ and Tissue Transplantation Services, Annex 1). The information text in the form referred to the approving body as the “Non-Interventional Clinical Research Ethics Committee”; its official name, as given in the approval letter and in the manuscript, is the Clinical Research Ethics Committee of the University of Health Sciences, Gazi Yaşargil Training and Research Hospital (decision no. 420, 28 August 2026). The form text is reproduced here as participants saw it.

PART I — ENGLISH TRANSLATION

Section 1. Information and consent

Dear colleague,

This survey has been prepared to assess the knowledge and decision-making approaches of anaesthesiology and reanimation residents with regard to the brain death determination process.

The survey takes approximately 4 minutes.

Participation is entirely voluntary. Your name, e-mail address or any information that could identify you is not collected. Your responses will be evaluated only in aggregate; they will not be reported at the level of the individual or the institution, and will under no circumstances be used for performance appraisal. Your individual score will not be reported to you or to anyone else.

You may abandon the survey at any stage. However, because the survey is fully anonymous, once submission is complete it will not be possible to determine which responses belong to which participant, and your data cannot be withdrawn.

This study examines decision-making processes; for this reason, some questions may be presented in different forms to different participants. Detailed information on this will be given at the end of the survey.

Approval for the study has been obtained from the Non-Interventional Clinical Research Ethics Committee of the University of Health Sciences, Gazi Yaşargil Training and Research Hospital.

For questions: [corresponding author’s e-mail address]

I have read the information above and agree to participate in the study voluntarily.
I agree | I do not agree (→ survey ends)

Section 2. A few questions about you

Your age
[open numeric response]

Your sex
Female | Male | Prefer not to state

Which year of residency training are you in?

1st year | 2nd year | 3rd year | 4th year | 5th year or later

Type of institution in which you work

University hospital | Training and research hospital | Other

Total duration of your intensive care rotation

I have not rotated | Less than 3 months | 3–6 months | More than 6 months

In how many brain death determination processes have you personally taken part to date?

None | 1–2 | 3–5 | More than 5

How many apnoea tests have you performed or taken part in?

None | 1–2 | 3–5 | More than 5

Have you received formal instruction (lecture, course or certified programme) on brain death?

Yes | No

Section 3. Knowledge statements

For each statement, please indicate both your answer and how confident you are in that answer within the same option.

Response options for all ten statements: True — I am confident | True — I am not confident | False — I am confident | False — I am not confident | I do not know

B1. Brain death can be determined only if the patient is an organ donor.

Keyed answer: False

B2. Under the legislation in force, the decision of brain death is made by two physicians: one a neurologist or neurosurgeon, and one a specialist in anaesthesiology and reanimation or in intensive care.

Keyed answer: True

B3†. In the assessment of brain death, the effect of neuromuscular blocking agents must be excluded; in case of doubt this should be confirmed with a nerve stimulator (train-of-four).

Keyed answer: True

B4†. A brainstem examination performed in a patient with a mean arterial pressure of 50 mmHg is considered valid for the determination of brain death.

Keyed answer: False

B5†. In anoxic brain injury following cardiopulmonary resuscitation, the neurological examination for brain death may be started immediately.

Keyed answer: False

B6. In adults, at least 12 hours must elapse between the first and second neurological examinations.

Keyed answer: True

B7†. For the apnoea test to be considered consistent with brain death, $PaCO_2$ must rise to 60 mmHg or above, or increase by 20 mmHg or more from baseline.

Keyed answer: True

B8†. In a patient with chronic hypercapnia (for example advanced COPD), the apnoea test can be reliably interpreted using the standard threshold.

Keyed answer: False

B9. If the apnoea test cannot be performed for reasons such as pneumothorax, the determination may be completed with an ancillary test assessing cerebral circulation.

Keyed answer: True

B10†. EEG findings may be misleading in the presence of high-dose sedative drugs.

Keyed answer: True

Section 4. Clinical vignettes

Vignette 1†. 42-year-old man.
Being managed in the intensive care unit for severe head trauma.
Glasgow Coma Score 3; brainstem reflexes absent.
Oesophageal temperature 31.2 °C.
Haemodynamically stable.

Would you start the apnoea test at this stage?

Yes — I am confident | Yes — I am not confident | No — I am confident | No — I am not confident

Keyed answer: No

Vignette 2†. In a patient whose clinical examination is consistent with brain death, a flexion movement of the upper extremity is observed during application of a painful stimulus.

What would your approach be in the face of this finding?

The process continues; the finding is of spinal origin — I am confident | The process continues; the finding is of spinal origin — I am not confident | The determination process is halted — I am confident | The determination process is halted — I am not confident

Keyed answer: The process continues

Vignette 3†. 58-year-old patient, intracerebral haemorrhage.
Received a midazolam infusion for five days; the infusion was stopped 14 hours ago.
Creatinine 3.8 mg/dL; the patient is oliguric.

Would you start the clinical examination for brain death?

Yes — I am confident | Yes — I am not confident | No — I am confident | No — I am not confident

Keyed answer: No

Section 5. Randomised scenario (not scored)

61-year-old man, massive intracerebral haemorrhage.
On mechanical ventilation for 48 hours.
Sedation was stopped 36 hours ago.
Glasgow Coma Score 3; pupils fixed and dilated.
Cough and gag reflexes absent.
Haemodynamically stable on low-dose noradrenaline; the patient is normothermic.
Metabolic parameters within normal limits.

Closing sentence, version A: “The family has stated that they are favourably disposed to organ donation should the patient die.”

Closing sentence, version B: “The family has stated that they would definitely not consider organ donation should the patient die.”

Would you initiate the brain death determination process in this patient now?

Yes — I am confident | Yes — I am not confident | No — I am confident | No — I am not confident

When would you request a neurology or neurosurgery consultation?

Immediately | After 24 hours | I would not request one

Did the family’s attitude toward organ donation influence your decision?

1 — Not at all | 2 | 3 | 4 | 5 — Very much

Section 6. Your views

To what extent do you agree with the following statements? (1 Strongly disagree – 5 Strongly agree)

- I am concerned about the legal consequences of determining brain death.
- I feel competent to explain the concept of brain death to a family.

- I am uncomfortable that brain death determination and organ donation are intertwined.
- I feel prepared to serve on a brain death determination board once I am a specialist.
- I consider the instruction I received on this subject during my residency adequate.

What barriers have you encountered, or do you think you may encounter, in the brain death determination process? (More than one option may be selected.)

Legal concern | Inadequate training | Lack of supervision | Time and workload | Difficulty communicating with the family | Anxiety about forensic proceedings | Absence of a written protocol in the institution | I see no barriers

Section 7. Final two questions

Where did you mainly acquire your knowledge on this subject?

Residency lectures | By observing senior physicians | Congress or course | My own reading | I have had no formal source

Does your institution have a written brain death protocol?

Yes | No | I do not know

Debriefing text (shown to all participants after submission)

Thank you very much for your participation.

The aim of this study is to assess not only the level of knowledge about the brain death determination process, but also the extent to which confidence in that knowledge corresponds to accuracy.

In the final clinical scenario of the survey, some participants were told that the patient’s relatives were favourably disposed to organ donation, and others that they were opposed. The patient’s medical condition was exactly the same in both groups. This variation was made in order to measure whether the decision concerning the determination process is influenced by a non-medical contextual variable, and could not be disclosed in advance for the sake of the validity of the study.

It must be emphasised that brain death is determined solely according to medical criteria, entirely independently of the possibility of organ donation.

Under the legislation in force, the decision of brain death is made unanimously by two physicians: one a neurologist or neurosurgeon, and the other a specialist in anaesthesiology and reanimation or in intensive care.

If you would like to receive information about the results of the study, you may contact [corresponding author’s e-mail address].

PART II — TURKISH ORIGINAL (as seen by participants)

Bölüm 1. Bilgilendirme ve onam

Sayın meslektaşım,

Bu anket, anesteziyoloji ve reanimasyon uzmanlık öğrencilerinin beyin ölümü tanı sürecine ilişkin bilgi ve karar verme yaklaşımlarını değerlendirmek amacıyla hazırlanmıştır.

Anket yaklaşık 4 dakika sürmektedir.

Katılım tamamen gönüllüdür. Adınız, e-posta adresiniz veya sizi tanımlayabilecek herhangi bir bilgi toplanmamaktadır. Yanıtlarınız yalnızca toplu olarak değerlendirilecek; kişi veya kurum bazında raporlanmayacak, hiçbir şekilde performans değerlendirmesi amacıyla kullanılmayacaktır. Bireysel puanınız size veya başka bir kişiye bildirilmeyecektir.

Anketi istediğiniz aşamada yarıda bırakabilirsiniz. Ancak anket tümüyle anonim olduğu için, gönderim tamamlandıktan sonra yanıtlarınızın hangi katılımcıya ait olduğu belirlenemeyecek ve verileriniz geri çekilemeyecektir.

Bu çalışma karar verme süreçlerini incelemektedir; bu nedenle bazı sorular katılımcılar arasında farklı biçimlerde sunulabilmektedir. Anketin sonunda bu konuda ayrıntılı bilgi verilecektir.

Çalışma için Sağlık Bilimleri Üniversitesi Gazi Yaşargil Eğitim ve Araştırma Hastanesi Girişimsel Olmayan Klinik Araştırmalar Etik Kurulu'ndan onay alınmıştır.

Sorularınız için: [sorumlu yazarın e-posta adresi]

Yukarıdaki bilgilendirmeyi okudum ve çalışmaya gönüllü olarak katılmayı kabul ediyorum.
Kabul ediyorum | Kabul etmiyorum

Bölüm 2. Sizinle ilgili birkaç soru

Yaşınız
[açık uçlu sayısal yanıt]

Cinsiyetiniz
Kadın | Erkek | Belirtmek istemiyorum

Uzmanlık eğitiminizin kaçıncı yılındasınız?
1. yıl | 2. yıl | 3. yıl | 4. yıl | 5. yıl ve üzeri

Görev yaptığınız kurum tipi
Üniversite hastanesi | Eğitim ve araştırma hastanesi | Diğer

Toplam yoğun bakım rotasyon süreniz
Rotasyon yapmadım | 3 aydan az | 3–6 ay | 6 aydan fazla

Bugüne kadar kaç beyin ölümü tanı sürecinde bizzat bulundunuz?
Hiç | 1–2 | 3–5 | 5'ten fazla

Kaç apne testi uyguladınız veya uygulanmasına katıldınız?
Hiç | 1–2 | 3–5 | 5'ten fazla

Beyin ölümü konusunda formal bir eğitim (ders, kurs veya sertifikalı program) aldınız mı?
Evet | Hayır

Bölüm 3. Bilgi soruları

Her ifade için hem yanıtınızı hem de bu yanıttan ne kadar emin olduğunuzu aynı seçenekte belirtiniz.

On ifadenin tümü için yanıt seçenekleri: Doğru — eminim | Doğru — emin değilim | Yanlış — eminim | Yanlış — emin değilim | Bilmiyorum

B1. Beyin ölümü tanısı konulabilmesi için hastanın organ bağışçısı olması gerekir.

B2. Yürürlükteki mevzuata göre beyin ölümü kararı; biri nörolog veya nöroşirürjiyen, biri anesteziyoloji ve reanimasyon veya yoğun bakım uzmanı olmak üzere iki hekim tarafından verilir.

B3. Beyin ölümü değerlendirmesinde nöromüsküler blokör etkisinin dışlanması gereklidir; şüphe halinde sinir stimülatörü (TOF) ile doğrulanmalıdır.

B4. Ortalama arter basıncı 50 mmHg olan bir hastada yapılan beyin sapı muayenesi, beyin ölümü tanısı için geçerli kabul edilir.

B5. Kardiyopulmoner resüsitasyon sonrası gelişen anoksik beyin hasarında, beyin ölümü nörolojik muayenesine hemen başlanabilir.

B6. Erişkinde birinci ve ikinci nörolojik muayene arasında en az 12 saat geçmelidir.

B7. Apne testinin beyin ölümü ile uyumlu kabul edilmesi için $PaCO_2$ değerinin 60 mmHg ve üzerine çıkması veya bazal değere göre 20 mmHg ve üzerinde artması gerekir.

B8. Kronik hiperkapnisi olan bir hastada (örneğin ileri evre KOAH) apne testi, standart eşik değer kullanılarak güvenle yorumlanabilir.

B9. Pnömotoraks gibi nedenlerle apne testi uygulanamıyorsa, beyin dolaşımını değerlendiren bir destekleyici test ile tanı tamamlanabilir.

B10. EEG bulguları, yüksek doz sedatif ilaç varlığında yanıltıcı olabilir.

Bölüm 4. Klinik vinyetler

Vinyet 1. 42 yaşında erkek hasta.
Ağır kafa travması nedeniyle yoğun bakımda izleniyor.
Glasgow Koma Skoru 3, beyin sapı refleksleri alınamıyor.
Özofageal ısı 31,2 °C.
Hemodinamik olarak stabil.

Bu aşamada apne testine başlar mısınız?

Evet — eminim | Evet — emin değilim | Hayır — eminim | Hayır — emin değilim

Vinyet 2. Klinik muayenesi beyin ölümü ile uyumlu bir hastada, ağrılı uyaran uygulanması sırasında üst ekstremitede fleksiyon hareketi gözleniyor.

Bu bulgu karşısında yaklaşımınız ne olurdu?

Süreç devam eder; bulgu spinal kökenlidir — eminim | Süreç devam eder; bulgu spinal kökenlidir — emin değilim | Tanı süreci durdurulur — eminim | Tanı süreci durdurulur — emin değilim

Vinyet 3. 58 yaşında hasta, intraserebral kanama.
Beş gün boyunca midazolam infüzyonu almış, infüzyon 14 saat önce kesilmiş.
Kreatinin 3,8 mg/dL, hasta oligürik.

Beyin ölümü klinik muayenesine başlar mısınız?

Evet — eminim | Evet — emin değilim | Hayır — eminim | Hayır — emin değilim

Bölüm 5. Randomize senaryo

61 yaşında erkek hasta, masif intraserebral kanama.
48 saattir mekanik ventilasyon desteğinde.
Sedasyon 36 saat önce kesildi.
Glasgow Koma Skoru 3, pupiller fiks ve dilate.
Öksürük ve öğürme refleksi alınamıyor.
Hemodinami düşük doz noradrenalin ile stabil, hasta normotermik.

Metabolik parametreler normal sınırlarda.

Son cümle, A versiyonu: “Aile, hasta kaybedilirse organ bağışına sıcak baktığını ifade etmiştir.”

Son cümle, B versiyonu: “Aile, hasta kaybedilirse organ bağışını kesinlikle düşünmediğini ifade etmiştir.”

Bu hastada beyin ölümü tanı sürecini şimdi başlatır mıydınız?

Evet — eminim | Evet — emin değilim | Hayır — eminim | Hayır — emin değilim

Nöroloji veya nöroşirürji konsültasyonunu ne zaman isterdiniz?

Hemen | 24 saat sonra | İstemezdim

Kararınızda ailenin organ bağışına yaklaşımı etkili oldu mu?

1 — Hiç etkili olmadı | 2 | 3 | 4 | 5 — Çok etkili oldu

Bölüm 6. Görüşleriniz

Aşağıdaki ifadelere ne ölçüde katılıyorsunuz? (1 Kesinlikle katılmıyorum – 5 Kesinlikle katılıyorum)

- Beyin ölümü tanısı koymanın yasal sonuçlarından endişe duyarım.
- Aileye beyin ölümü kavramını açıklamakta kendimi yeterli hissediyorum.
- Beyin ölümü tanısı ile organ bağışı konularının iç içe geçmesi beni rahatsız ediyor.
- Uzman olduğumda beyin ölümü kurulunda görev almaya kendimi hazır hissediyorum.
- Uzmanlık eğitimim sırasında bu konuda aldığım eğitimi yeterli buluyorum.

Beyin ölümü tanı sürecinde karşılaştığınız veya karşılaşabileceğinizi düşündüğünüz engeller nelerdir? (Birden fazla seçenek işaretleyebilirsiniz.)

Yasal endişe | Yetersiz eğitim | Süpervizyon eksikliği | Zaman ve iş yükü | Aileyle iletişim güçlüğü | Adli süreç kaygısı | Kurumda yazılı protokol bulunmaması | Engel görmüyorum

Bölüm 7. Son iki soru

Bu konudaki bilgilerinizi ağırlıklı olarak nereden edindiniz?

Uzmanlık eğitimi dersleri | Kıdemli hekimleri gözlemleyerek | Kongre veya kurs | Kendi okumalarım | Formal bir kaynağım olmadı

Kurumunuzda yazılı bir beyin ölümü protokolü var mı?

Evet | Hayır | Bilmiyorum

Bilgilendirme metni (gönderim sonrası tüm katılımcılara gösterildi)

Katılımınız için çok teşekkür ederiz.

Bu çalışmanın amacı, beyin ölümü tanı sürecine ilişkin bilgi düzeyinin yanı sıra, bu bilgiye duyulan güvenin doğrulukla ne ölçüde örtüştüğünü değerlendirmektir.

Ankette yer alan son klinik senaryoda, katılımcıların bir bölümüne hasta yakınlarının organ bağışına olumlu, diğer bölümüne ise olumsuz baktığı bilgisi sunulmuştur. Her iki grupta hastanın tıbbi durumu tamamen aynıydı. Bu farklılaştırma, tanı sürecine ilişkin kararın tıbbi olmayan bir bağlam değişkeninden etkilenip etkilenmediğini ölçmek amacıyla yapılmıştır ve çalışmanın geçerliliği açısından önceden bildirilememiştir.

Önemle belirtmek gerekir ki beyin ölümü tanısı, organ bağışı olasılığından tamamen bağımsız olarak, yalnızca tıbbi ölçütlere göre konulur.

Yürürlükteki mevzuata göre beyin ölümü kararı, biri nörolog veya nöroşirürjiyen, diğeri anesteziyoloji ve reanimasyon veya yoğun bakım uzmanı olmak üzere iki hekim tarafından oy birliği ile verilir.

Çalışmanın sonuçları hakkında bilgi almak isterseniz [sorumlu yazarın e-posta adresi] üzerinden

iletişime geçebilirsiniz.
